# Unraveling the complex interplay between anti-tumor immune response and autoimmunity mediated by B cells and autoantibodies in the era of anti-checkpoint monoclonal antibody therapies

**DOI:** 10.3389/fimmu.2024.1343020

**Published:** 2024-01-22

**Authors:** Sarah Soussan, Guilhem Pupier, Isabelle Cremer, Pierre-Emmanuel Joubert, Catherine Sautès-Fridman, Wolf Herman Fridman, Sophie Sibéril

**Affiliations:** Centre de recherche des Cordeliers, INSERM U1138, Sorbonne Université, Université Paris Cité, Paris, France

**Keywords:** B cells, autoantibodies, cancer, autoimmunity, immune checkpoints, therapeutic monoclonal antibodies

## Abstract

The intricate relationship between anti-tumor immunity and autoimmunity is a complex yet crucial aspect of cancer biology. Tumor microenvironment often exhibits autoimmune features, a phenomenon that involves natural autoimmunity and the induction of humoral responses against self-antigens during tumorigenesis. This induction is facilitated by the orchestration of anti-tumor immunity, particularly within organized structures like tertiary lymphoid structures (TLS). Paradoxically, a significant number of cancer patients do not manifest autoimmune features during the course of their illness, with rare instances of paraneoplastic syndromes. This discrepancy can be attributed to various immune-mediated locks, including regulatory or suppressive immune cells, anergic autoreactive lymphocytes, or induction of effector cells exhaustion due to chronic stimulation. Overcoming these locks holds the risk to induce autoimmune mechanisms during cancer progression, a phenomenon notably observed with anti-immune checkpoint therapies, in contrast to more conventional treatments like chemotherapy or radiotherapy. Therefore, the challenge arises in managing immune-related adverse events (irAEs) induced by immune checkpoint inhibitors treatment, as decoupling them from the anti-tumor activity poses a significant clinical dilemma. This review summarizes recent advances in understanding the link between B-cell driven anti-tumor responses and autoimmune reactions in cancer patients, and discusses the clinical implications of this relationship.

## Introduction

The tumor microenvironment (TME) is a dynamic and complex ecosystem where various immune cells play crucial roles. Among these, tumor-infiltrating B cells (TIL-B cells) exert a variety of functions in the TME (1) such as local secretion of antibodies of different isotypes (mainly IgM, IgG and IgA) that can recognize tumor cells (2, 3). An association has early been established between autoimmunity and cancer, as evidenced by the frequent presence of serum autoantibodies in cancer patients, which have subsequently been used as biomarkers for anti-tumor responses (4). However, the link between autoreactive serum antibodies and anti-tumor B-cell responses, has been largely unexplored. Furthermore, there is a lack of understanding regarding the association between these autoantibodies and the occurrence of autoimmunity in cancer patients, as well as the temporal relationship between autoimmune disorders and the development of anti-tumor immune responses. Nevertheless, the association between the two pathologies is currently under reevaluation due to the emerging issue of immune-related adverse events (irAEs) associated with the use of immunotherapies that no longer target the tumor cell directly but instead modulate the immune system (5).

In this review, we underline that most tumor antigens recognized by serum antibodies or tumor-infiltrating B cells identified so far are modified or unmodified self-antigens, with many of them being intracellular. We will propose several mechanisms enabling autoantibodies to access these intracellular antigens. We will also discuss the connection between autoimmunity and anti-tumor responses in cancer patients, specifically focusing on the humoral responses against tumors, that arise within the tumor microenvironment (TME) in well-organized structures such as tertiary lymphoid structures (TLS) (6). The breakdown of tolerance induced by inflammatory condition within the TME raises questions concerning the emergence of broader autoimmune symptoms in cancer patients, the temporal and causal link between autoimmune and anti-tumor responses, and the impact of anti-cancer therapies, particularly immunotherapies, on this relationship. We delve into this issue, specifically in patients treated with monoclonal antibodies (mAbs) targeting inhibitory anti-PD-1 and anti-CTLA-4 immune checkpoints. The emergence of acute or chronic side effects associated with the increasing use of these therapies is indeed a major clinical concern in patient’s management. Finding therapeutic strategies to predict or manage these side effects without compromising the efficacy of anti-tumor therapy is therefore a tremendous challenge.

## Antibodies directed against self-antigens: intersecting effectors of cancer immunity and autoimmunity

Hematological malignancies and solid tumors are associated with the induction of autoimmunity that is characterized by the generation of intratumor or circulating autoantibodies against a wide range of antigens (7, 8). Different approaches have been applied for the identification of serum autoantibody profiles in various cancers, including SEREX (serological identification of antigens by screening of cDNA expression libraries derived from tumors), conventional high-throughput proteomics techniques (*i.e.* 2-D gel electrophoresis followed by mass spectrometry), human proteome microarrays, or use of phage-displayed peptide library to capture individual-specific IgG antibody repertoires combined with high-throughput sequencing to uncover captured peptides (9). Similarly, recent studies have delved into the characterization of antigens recognized by anti-tumoral B cells. However, this issue poses significant technical challenges involving high-throughput isolation of single B cells (including plasma cells or memory/effector B cells) from the blood or tumor tissue of cancer patients, sequencing of immunoglobulin genes, generation of mAbs libraries, and screening of antigens recognized by these mAbs (2, 10).

It has been demonstrated in some studies that humoral immune responses in cancer patients may target neo-antigens - derived from frameshift or replacement mutation, alternative start codon or alternative splicing events (11–13)-, as well as endogenous retroviruses-derived antigens (14) or papilloma virus antigens (15). Nevertheless, most of cancer-related antibodies described so far recognize self-antigens (8), also expressed by healthy cells. These anti-tumor autoantibodies may be directed against oncoproteins, tumor suppressor proteins, or proliferation associated proteins (7, 16) and, interestingly, the repertoire of autoantibodies found in cancer patients overlaps to a significant extent with that of patients with autoimmune diseases [Anti-nuclear antibodies (ANAs), anti-p53, anti-phospholipids, anti-cytokeratin, anti-c-myc….)] (17–19). Autoantibodies in cancer patients may have anti-tumor effects, by blocking the interactions between growth receptors and their ligands, targeting key molecules implicated in apoptosis/viability signaling pathways, or by interfering with adhesion molecules expressed by tumor cells (inhibiting metastases formation). As an example, endogenous antibodies directed against HER2/neu receptor, identified in cancer patients (20–22), can suppress HER2 phosphorylation and downstream activation of ERK and inhibit the transformed phenotype of HER2-expressing tumor cells (23). Moreover, ANAs immune complexes could have direct or indirect anti-tumor effects either by inducing cytotoxicity (ADCC) or by mediating the clearance of extracellular nuclear chromatin -released from apoptotic cancer cells- that inhibits natural killer (NK) cell activity (18, 19). It has also been suggested that anti-DNA autoantibodies, and more generally autoantibodies with the ability to penetrate into the nucleus, can impact DNA synthesis, repair or transcription mechanisms, potentially leading to apoptotic or anti-proliferative effects (24–26). Nevertheless, it should be mentioned that, autoreactive antibodies are not always associated with anti-tumor effector function but can be pro-tumoral. In fact, certain antigenic specificities of serum antibodies have been correlated with poor prognosis, such as p53 (27).

**Figure 1:**
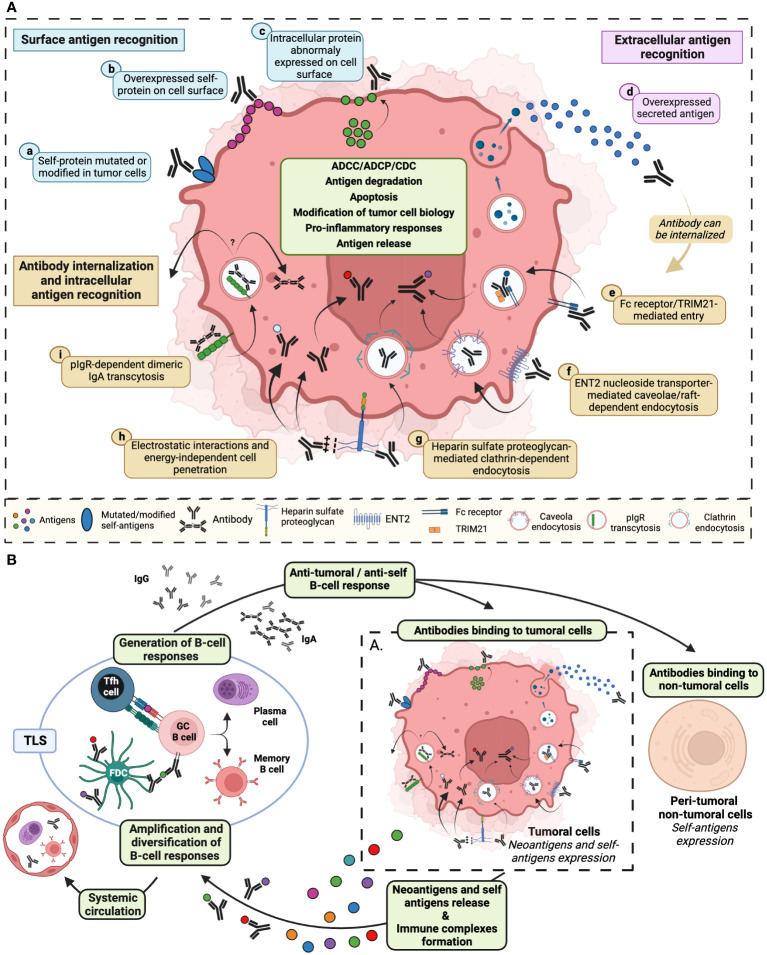
Diverse mechanisms of tumor cell self-antigens recognition by antibodies stimulate both anti-tumor and anti-self B-cell responses. **(A)** Anti-tumor antibodies can be directed against: surface antigens (blue) such as (a) modified or mutated self-proteins expressed on tumor cell membrane, (b) self-proteins overexpressed on the cell surface, (c) intracellular proteins abnormally exposed to the tumor cell surface; extracellular antigens (pink) such as d) self-proteins secreted in the tumor microenvironment; intracellular self-antigens recognized following antibody internalization, via (e) antibody interaction with cell surface Fc receptor and/or with TRIM21 cytosolic Fc receptor, (f) antibody fixation to ENT2 protein facilitating antibody cell penetration through calveolae/raft-dependent endocytosis, (g) antibody recognition of proteoglycan that initiates clathrin-dependent endocytosis, (h) electrostatic interaction between antibody CDR3 enriched in basic amino acids and negatively charged cell surfaces that could lead to passive internalization, (i) the binding of dimeric IgA to pIgR resulting in transcytosis initiation. After internalization, encapsulated antibodies may recirculate within the tumor microenvironment, transit to the opposite pole of the cell via transcytosis, escape from the endocytic vesicle to the cytosol, or migrate to the nucleus. Antibodies binding to neoantigens or self-antigens may result in the recruitment of immune cells allowing tumor cell elimination through ADCC, ADCP and CDC. Antibody internalization could also induce degradation of recognized proteins, tumor cell apoptosis, release of pro-inflammatory cytokines, modification of intracellular processes and release of self-antigens. **(B)** These effector mechanisms are prone to induce and amplify, in tertiary lymphoid structures (TLS) or in the tumor draining lymph node, a B-cell dependent immunity specific to tumor- and self-proteins. *De novo* generated anti-tumoral and anti-self antibodies may bind either to tumor cells or to non-tumoral cells, initiating a new amplification loop. Locally induced self-reactivity (B cells and antibody) can recirculate through the systemic circulation and be detected in peripheral blood. ADCC, antibody-dependent cellular cytotoxicity; ADCP, antibody-dependent cellular phagocytosis; CDC, complement-dependent cytotoxicity; ENT2, equilibrative nucleoside transporter 2; pIgR, polymeric immunoglobulin receptor; TRIM21, tripartite motif-containing protein 21. Created with BioRender.com.

Since a large proportion of anti-tumor antibodies recognize intracellular self-antigens (28, 29), the mechanism by which the intracellular localization of tumor targets enables an efficient anti-tumor activity of such antibodies remains an enigma. The highly inflammatory and fibrotic conditions within the TME may result in the surface-expression of certain intracellular antigens on tumor cells. This renders these antigens accessible to the autoantibodies that specifically target them (2, 30) (**Figure 1A**). Several mechanisms involving an internalization phenomenon have also been proposed, notably thanks to studies conducted on autoantibodies found in autoimmune pathologies (31) (**Figure 1A**). The internalization process may involve the interaction of autoantibodies enriched in basic amino acids located in the complementarity-determining region 3 (CDR3) - such as arginine - with negatively charged cell-surface proteins, like heparin sulfate proteoglycan. These electrostatic interactions can be followed by either clathrin-dependent endocytosis (32) or energy-independent direct crossing of the plasma membrane (33) (**Figure 1A**). For the latter mechanism, elevated electrostatic interactions and potentially elevated concentration of antibodies could facilitate their internalization through transient pore formation or membrane destabilization, mechanisms that have been previously described for cell-penetrating peptides (CPP) (33, 34). Furthermore, the internalization can occur through Fc receptor/TRIM21-mediated entry (35, 36). Recent studies have also showed that IgA antibodies might enter tumor cells through a pIgR-dependent transcytosis mechanism (**Figure 1A**). This process hinders oncogenic signals, thereby enhancing the cytotoxic activity of T cells (37). Finally, an internalization of antibodies via caveolae/raft-dependent endocytosis of nucleoside transporter ENT2 has also been reported (38, 39) (**Figure 1A**). After the internalization, antibodies need to escape from endosomes along the endocytic pathway to reach the cytosol. It has been proposed that endosomal acidic pH induces conformational changes in the CDR3 of cytosol-penetrating antibody that leads to interactions with endosomal membranes. These interactions induce destabilization of endosomal membranes, subsequently leading to membrane pore formation and transport of antibodies to the cytosol (40) – a mechanism also described for CPP (34). Finally, it has also been suggested that increase in the concentration of vesicle contents might enhance endosomal escape (34).

## The tumor microenvironment: a hub for induction of auto-reactive humoral immune responses

Whether autoreactivity that contributes to anti-tumor immunity is pre-existing or induced during spontaneous intra-tumoral humoral responses is not yet totally understood. Recently, a study by Mazor et al. delved into the origins of the anti-tumor specificity of intra-tumoral autoreactive B cells (2). The authors isolated single cells from fresh tumor samples and sequenced the genes encoding the variable regions of infiltrating IgG1^+^ antibody-secreting cells (ASCs). Subsequently, they cloned and expressed a repertoire of recombinant IgG1 antibodies derived from these cells. A significant fraction of these recombinant antibodies exhibited binding reactivity to primary ovarian cancer cells and/or to different pancreatic cancer cell lines, suggesting that antibodies derived from intra-tumor B cells target widely expressed “public antigens”. Indeed, the majority of the monoclonal recombinant antibodies recognized matrix metalloproteinase proteins (MMP), and in particular MMP14, a self-protein that is overexpressed in the inflammatory and fibrotic TME. To assess the impact of somatic hypermutation (SHM) on anti-tumor antibody specificity, the authors reverted the sequences encoding variable regions to their germline versions. Interestingly, the results showed that the pool of tumor-reactive antibodies is partly composed of antibodies that confer anti-tumor protection in their germline configurations, confirming earlier studies showing that such pre-existing natural autoantibodies can eliminate tumor cells in mice by binding to tumor antigens (41). However, another part of the tumor-reactive antibodies loses its binding capacity to cancer cells after germline reversion. Hence, the anti-tumor specificity of antibodies in ovarian cancer patients might also derive from intra-tumoral humoral response associated with acquired mechanisms of affinity maturation and selection of autoreactive B cells. In line with this study, baseline intra-tumor active B-cell responses characterized by autoimmune and polyreactive features in the TME have also been outlined in a recent study in melanoma patients (42). In this study, tumors displayed *in situ* SHM, clonal expansion, class switch recombination and elevated expression of RAG1 and RAG2 molecules indicating receptor editing. The authors also identified 32 potential autoantigens exclusively recognized by melanoma patient’ autoantibodies not found in healthy donors, with higher levels in active disease stages.

Thus, these different observations suggest the existence of a break of B-cell tolerance leading to the production of autoantibodies by B cells within the tumor. This failure of B-cell tolerance mechanisms can have various origins. Firstly, certain self-proteins identified as targets of adaptive immune responses in cancer exhibit spatial and/or temporal restrictions in their expression (like testis antigens, for instance), enabling their evasion from central and peripheral tolerance mechanisms. B-cell tolerance breakdown could also be induced by cancer-specific post-translational modifications or elevated expression of these proteins. Secondly, the immune system may be capable of sensing the phenotypic, functional and metabolic dysregulation of autologous cancer cells that occurs during tumorigenesis and cancer growth (41, 43). Indeed, modifications of peripheral autoreactivity profiles during tumorigenesis have been observed and the occurrence of certain autoantibodies has been found to precede clinical manifestations of cancer by several months to years, suggesting emergence of tumor immunogenicity at the very early stages of tumorigenesis (43). Interestingly, autoantibodies reactivity profiles against membrane, nuclear, nucleolar and cytoplasmic tumor-associated antigens have the potential to provide unique signatures that might reflect the nature or the stage of the malignant disease (43, 44). Thirdly, hyper-inflammation and components of innate immune system within the TME likely play significant roles in the initial stages that result in the loss of self-tolerance. Several studies, using either IFNAR1 knockout mice or anti-IFNAR1 blocking antibodies have highlighted the pivotal role of this signaling pathway, specifically in the priming of adaptive anti-tumor responses (45). Within the TME, a substantial release of nucleic acids from apoptotic cells may activate DNA or RNA sensing pathways such as toll-like receptors (TLR3, 7, 8, 9), cyclic GMP-AMP synthase (cGAS)/STING or RIG-I-like receptors/MAVS pathways. This activation would induce a robust expression of type I IFNs and strongly promote an adaptive anti-tumor response directed, at least in part, against tumor-derived autoantigens originating from these apoptotic cells (45). These events, relying on inflammation and the activity of innate cells, likely shape the local infiltration and organization of anti-tumor/anti-self adaptive immune cells.

Different studies using a plethora of techniques including multicolor or mass flow cytometry, multiplex immunofluorescence, bulk RNA sequencing, single-cell RNA sequencing or spatial transcriptomics (16) shed light on the abundance and variety of B-cell subsets found in the TME. In various cancers including melanoma, prostate cancer, colorectal cancer, lung adenocarcinoma, and breast cancer, memory B cells and plasma cells (PCs) dominate TIL-B infiltrates and elevated densities of these cells were observed within the TME compared to healthy tissue counterparts or peripheral blood (42, 46, 47). Additionally, a PC signature was linked to exceptionally long-term survival in ovarian cancer and other malignancies, emphasizing the potential prognostic and functional significance of these B cell subsets in cancer patients (48–50). Indeed, a meta-analysis of transcriptomic data from approximately 18,000 human tumors revealed that the presence of intra-tumoral plasma cells is one of the most significant positive prognostic factors for patient survival outcomes in various non-brain solid tumors, except for large-cell lung carcinoma (51). Importantly, the prognostic significance of B lymphocytes within the tumor – and, therefore, probably their optimal functionality- heavily relies on their organization within the TME, particularly their organization into TLS (1) (**Figure 1B**). These specialized immune structures play a pivotal role in shaping the anti-tumor immune response. The presence of well-organized B cell structures in the tumor suggests an active and functional immune response, which is associated with better prognosis in many cancer types (51). Recent studies including spatial transcriptomics showed that, while immature TLS lack germinal centers, hindering full B cell activation and often fostering immunosuppressive pathways, mature TLS - equipped with follicular helper T cells, germinal centers and follicular dendritic cells - facilitate B cell proliferation/maturation and PC formation, probably resulting in “in situ” anti-tumor antibody production (**Figure 1B**) (3, 52–54). It is also crucial to note a significant contrast regarding the exclusion of self -reactive B cells which occurs between secondary Lymphoid Organs (SLO) and TLS in autoimmune conditions where TLS are characterized by lack of exclusion of autoreactive B cells, resulting in the local production of autoantibodies. The cytokine-rich TLS environment, abundant in survival factors and potentially lacking expression of key checkpoints for screening self-reactive cells may favor local break of tolerance (55, 56).

## Deciphering an enigma: do cancer patients develop autoimmune disorders?

Hence, the observations that Abs produced in the TME are directed against self-non-mutated proteins rather than being restricted to tumor-specific antigens underline the close relationship between self-tolerance breakdown and anti-tumor immunity. However, Mazor et al. showed that ovarian cancer patients that develop self-reactive antibodies recognizing healthy tissues in addition to tumor cells, do not manifest autoimmune symptoms. Moreover, the authors did not observe an increased incidence of autoimmune diseases in a large retrospective study of patients with ovarian cancer over a follow up period of up to 15 years. The non-persistence over the long-term of autoantibodies in the serum of cancer patients and the deleterious impact of certain therapies such as chemotherapy on the production of antibodies by B lymphocytes are among the hypotheses proposed by the authors to explain this paradox (2).

In contrast, in certain patients with cancer-associated rheumatic diseases (such as dermatomyositis, polymyositis, vasculitis, and scleroderma), the kinetics of pathology onset indicates that the autoimmune manifestation could be a paraneoplastic syndrome induced by anti-tumor immune responses (7, 57). Approximatively 15-20% of dermatomyositis cases are associated with cancer, and among them, a notable proportion of patients receive a diagnosis of malignant disease months or even years before the onset of rheumatic diseases (7, 57). Such a temporal relation has also been reported for simultaneous vasculitis and cancers, or for breast cancer and scleroderma (7). The observation coming from different case reports, that resection of a malignant tumor without the use of corticosteroids can be followed by a complete remission of autoimmune disease symptoms (58, 59) is also in line with a paraneoplastic mechanism. It should also be noted that a particular autoantibody profile can be observed in patients with cancer-associated dermatomyositis or scleroderma, distinct from the autoreactivity pattern classically found in these types of autoimmune diseases (57). This observation suggests that, in these patients, autoimmune responses implicated in myositis and scleroderma are likely to be associated with anti-tumor responses. Furthermore, Joseph et al. showed that CD4^+^ T cells and antibodies recognizing similarly a tumor neo-antigen derived from somatic mutations of the self-antigen RCP1 (the large subunit of RNA polymerase 3) and its wild type form can be detected in patients with cancer-associated scleroderma (60). Thus, the authors suggested that cancer-associated autoimmunity may also reflect an immune response initiated by mutated tumor antigens, that could be spread to the wild type autoantigen, inducing broader autoimmune reactivity.

**Figure 2:**
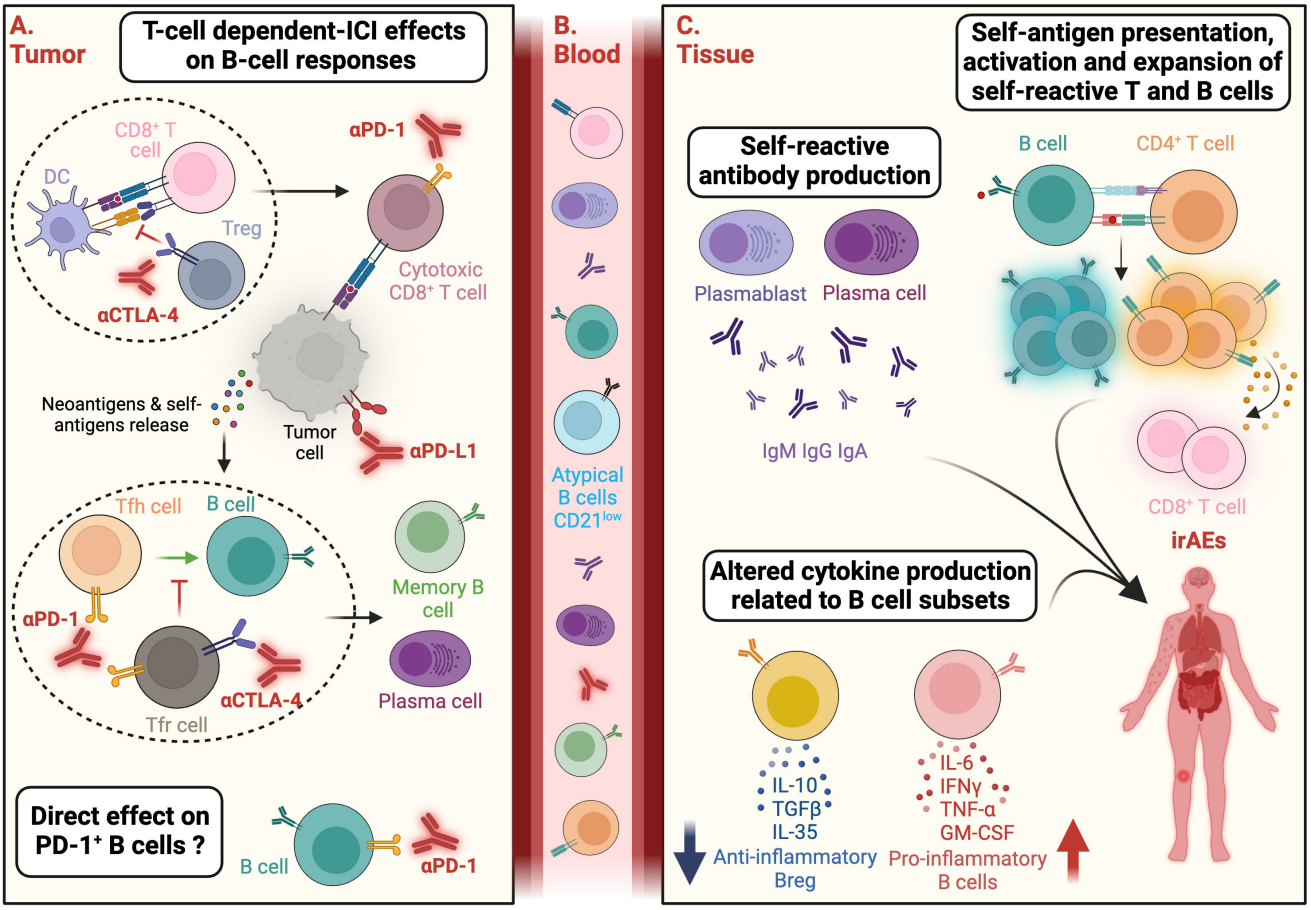
ICI-mediated direct and indirect activation of autoreactive B cells and potential functions associated with the onset of irAEs. **(A)** Immune checkpoint inhibitors **(ICI)** can activate autoreactive B lymphocytes through T-cell dependent mechanisms. ICI can have an impact on different T-cell subsets including regulatory T cells (Treg) and PD1^+^ CD8^+^ T cells (exhausted T cells) leading to an increase of the activation of anti-tumor CD8^+^ T cells. This activation results in tumor cell death, release of neoantigens and/or self-antigens. In addition, by modulating PD-1^+^ T follicular helper (Tfh) or PD1^+^ CTLA-4^+^ T follicular regulatory (Tfr) subsets, anti-CTLA-4 and anti-PD-1 mAbs can stimulate B cells activation and differentiation into memory B cells and plasma cells within germinal centers. Additionally, ICI may directly impact B-cell subsets expressing inhibitory immune checkpoints such as PD-1. **(B)** Activated B and T cells may subsequently circulate in the peripheral blood and participate to immune-related adverse events (irAEs) onset. In patients developing irAEs, the frequency of B-cell subsets enriched in autoreactive clones, such as the atypical CD21^low^ B-cell subset, can be increased in the peripheral blood. **(C)** At distance from the tumor tissue, B-cell functions implicated in irAE development might involve: i) self-reactive antibody production by antibody-secreting plasmablasts and plasma cells; ii) CD4^+^ T cell and B cell cooperation involving self-antigens presentation by B cells, activation and expansion of self-reactive T (CD4^+^ and CD8^+^) and B cells, iii) Altered ratio of pro-inflammatory cytokines (IL-6, TNFα, IFNγ, GM-CSF) produced by activated B cells and anti-inflammatory cytokines (IL-10, TGFβ, IL-35) produced by regulatory B cells. Created with BioRender.com.

Therefore, the question arises regarding the factors influencing the transition from local autoimmunity associated with the production of anti-tumor autoantibodies and driven by heightened expression of specific self-antigens within a pro-inflammatory TLS environment, towards a broader potentially systemic autoimmunity that extends beyond the tumor site (**Figure 1B**). Various peripheral inhibitory mechanisms, such as anergy, exhaustion of effector cells, and the presence of regulatory immune cells -such as Treg, regulatory follicular T cells (Tfr), regulatory B cells (Breg) or suppressive myeloid cells- (**Figure 2**), may act as a lockdown to mitigate autoimmune disorders in the context of cancer.

However, the use of therapies aiming to subvert these inhibitory mechanisms, such as immunogenic chemotherapies and interferon (IFNα), is associated with an increased incidence of autoimmune manifestations (61, 62). A particularly enlightening scenario that unveils the intricate connection between autoimmunity and anti-tumor responses is observed with the use of immune checkpoint inhibitors (ICI) targeting PD-1 and CTLA-4 inhibitory molecules (**Figure 2**). These inhibitors serve as a noteworthy case study, shedding light on the complex interplay between autoimmune reactions and the efficacy of anti-tumor responses.

## When anti-cancer therapy impacts underlying autoimmunity in cancer patients: the specific case of immune checkpoint inhibitors and immune-related adverse events

Immune checkpoints molecules consist of several stimulatory and inhibitory cell surface receptors that are critical for the regulation of immune cell functions. Under normal conditions, they are implicated in the initiation, the intensity and the duration of immune responses, and thus are critical for the prevention of autoimmune diseases. In preclinical mouse models, inhibition or genetic deletion of CTLA-4 and PD-1 leads to autoimmune disorders (63). Indeed, genetic deletion of CTLA-4 results in lymphoproliferation, multiorgan autoimmunity and early death in mice (64). Deletion of PD-1 or PD-L1 in mice results in various autoimmune manifestations ranging from arthritis to delayed cardiomyopathy which correlates with the loss of tolerance in the periphery (65, 66). In the last years, therapeutic mAbs directed against inhibitory immune checkpoints have changed the landscape of cancer therapy. Numerous clinical studies have demonstrated that such antibodies could induce durable clinical responses even in patients with advanced cancer (67).

Among the various immune checkpoints, CTLA-4, as well as the PD-1 pathways and its ligands (PD-L1 and PD-L2) have been most intensely studied. CTLA-4, expressed on T cells, is an important and early contributor to the development of immune tolerance. This molecule negatively regulates the priming and early antigen-dependent T-cell activation in the lymphoid organs, and is also expressed on Treg. PD-1 is an inhibitory checkpoint of T-cell activity within the peripheral tissues and the TME. PD-1 is also highly expressed on intra-tumoral Treg cells and may enhance the immunosuppressive activity of these cells. Thus, anti-CTLA-4 and anti-PD-1 mAbs restore anti-tumor activity by facilitating the differentiation or the reactivation of tumor-reactive cytotoxic CD8^+^ T cells and of helper CD4^+^ T cells. ICI mAbs potentialize the production of IFNγ and induce a broadening of T-cell repertoire, resulting in an increased frequency of anti-tumor clones (68). It has also been shown that ICI mAbs have an impact on the activation of effector T cells not expressing PD-1 or CTLA-4. Indeed, ICI have bystander effects on other innate and adaptive cells leading to a highly inflammatory context (68).

The first ICI mAb to receive an FDA approval was ipilimumab (anti-CTLA-4 mAb) in 2011 for the treatment of advanced stage melanoma (68). Since then, about 50% of all patients with metastatic cancer are eligible to receive ICIs. In September 2023, eleven anti-PD-1/PD-L1/CTLA-4 mAbs have been approved in US and/or EU for the treatment of different cancers. While both CTLA-4 and PD-1 mAbs have resulted in increased patient survival in numerous cancer types such as melanoma or non-small cell lung cancer (NSCLC), ICI still include important limitations. Notably, only 15-60% of treated patients seem to respond to ICI treatment (69). Furthermore, ICI treatment can also cause a spectrum of toxicities and adverse events that are mostly immune-related (irAEs). IrAEs, less commonly encountered in conventional cancer treatments such as radiotherapy or chemotherapy, include rash, thyroiditis, hypophysitis, adrenal insufficiency, pneumonitis, pancreatitis, colitis, autoimmune hepatitis, renal failure, and cardiotoxicity (70, 71). As expected from the varying mechanisms of action of anti-PD1 and anti-CTLA-4 mAbs, which act on distinct lymphocyte subtypes and at different sites, their concomitant use results in both a higher incidence, and a broader spectrum of adverse events, compared to the monotherapy groups (30-90% for anti-CTLA-4 mAb, 30-70% for anti-PD-1 mAb and up to 90% for anti-CTLA-4 mAb combined to anti-PD-1 mAb) (70).The immune mechanisms underlying irAEs have not been completely elucidated and rely probably on different pathways depending on the type of ICI. The direct binding of mAbs to the tissue has been proposed as a mechanism for some irAEs. For instance, CTLA-4 was demonstrated to be expressed at the surface of the pituitary gland cells (72, 73), and direct antibody binding leads to antibody-dependent cell cytotoxicity (ADCC) and complement-dependent cytotoxicity (CDC) which mediate inflammation and destruction of the tissue (72, 73). Furthermore, in patients treated with anti-CTLA-4 mAb, sequencing of TCRs from cytotoxic T cells showed an expansion of T cell clones that correlated with irAEs development (74). These results suggest that ICIs could lead to a diverse pool of T cells reactive against tumor antigens but also self-antigens (75, 76). A recent study also showed an association between early expansion of Ki-67^+^ regulatory T cells and Ki67^+^ CD8^+^ T cells and increased risk of irAES in ICI-treated NSCLC and melanoma patients (77). Remarkably, while several recent studies demonstrated that tumor infiltrating or circulating B cells and T follicular helper (Tfh)-like subsets are associated with response to ICI treatment and survival (54, 78–81), the role of B cells and their predictive value in irAEs has been less studied. Interestingly, modification in both TCR and BCR repertoire diversity has been correlated with progression-free survival but also with the occurrence of adverse events (82). Moreover, transcriptomic analyses performed on whole blood samples from advanced melanoma patients treated with ipilimumab (anti-CTLA-4) mAb, showed that the expression of several immunoglobulin genes increased throughout the treatment and that this increase was more prominent in the group of patients exhibiting gastrointestinal irAEs (83).

Alteration in the B cell compartment was recently observed in cancer patients undergoing ICI-induced irAEs. First, functional and quantitative defects in the B-regulatory cell repertoire at baseline were highlighted in ICI-treated patients with advanced-stage NSCLC (84). Moreover, a marked increase in class-switched memory B cells, plasmablasts and CD21^low^ B cells was observed after the first cycle of combined administration of anti-PD-1 and anti-CTLA-4 mAbs in melanoma patients (85) (**Figure 2**). This expansion preceded and correlated with both the frequency and timing of iRAEs. Of note, CD21^low^ B-cell subset is enriched in autoreactive clones and is expanded in patients with autoimmune diseases (86, 87). Another study conducted in a smaller number of patients with metastatic renal cell carcinoma also confirmed a significant increase of CD21^low^ B-cell subset in patients developing irAEs (88). Indeed, certain subsets of B cells -including CD21^low^ B cells - express PD-1 and/or CTLA-4, suggesting a direct effect of ICI on B-cell activation and function (89–96). Nevertheless, an indirect effect of anti-PD-1 and anti-CTLA-4 mAbs, mediated by T-Follicular Tfr and Tfh subsets should not be excluded (97) (**Figure 2**). Thus, the aforementioned data support the dysregulation of particular B-cell subsets at baseline or during treatment as markers of irAEs occurrence. However, these studies rely on a limited number of patients and lack of mechanistic data on the functions of B cells involved in ICI-induced toxicities (i.e., production of deleterious autoantibodies, of pro-inflammatory cytokines, modulation of T-cell activity, or complement system activation) (**Figure 2**).

## Decoupling anti-tumor effects of ICI from irAEs? a clinical challenge for optimizing treatment management and patient’ quality of life

As mentioned in this review, autoimmune response and anti-tumoral immunity are closely interconnected in cancer patients, raising questions about the relationship between irAEs and therapy response in the context of ICI therapies. IrAEs can be considered as markers for effective ICI-induced immunogenicity that may lead to immune responses both clinically favorable against the tumor and unfavorable against healthy tissues. Indeed, although some investigations have yielded mixed results regarding the utility of irAEs as prognostic indicator of treatment response, several studies observed that the occurrence of irAEs during ICI treatment could be positively correlated with disease control rate, overall survival and progression free survival (98, 99). Notably, the survival benefit for patients who developed irAEs was predominantly observed in patients presenting endocrinal (hypophysitis and thyroid dysfunction) and dermatological (rash, vitiligo) abnormalities (99–101). This association between irAEs and ICI efficacy could be explained mechanistically since an efficient ICI-induced anti-tumor immune response within the TME leads to tumor cell death associated with massive release of antigens that can activate autoreactive T cells and B cells. In fact, several studies strongly support the hypothesis that some of the released antigens might be shared between the tumor and organs affected by irAEs. Firstly, comparison of irAEs from three tumor types usually treated by anti-PD-1 mAbs, *i.e*. melanoma, NSCLC, and renal cell carcinoma (RCC), showed that melanoma patients had a higher frequency of gastrointestinal and skin irAEs and lower frequency of pneumonitis compared with NSCLC. Moreover, arthritis and myalgia were more common in melanoma patients compared with RCC where pneumonitis and dyspenoa were more prevalent (102). Thus, this study suggests that different immune microenvironments may drive organ-specific irAEs patterns. In line with this assumption, a prospective cohort study involving patients with NSCLC developing skin toxic effects post-ICI treatment, has allowed the identification of nine T-cell antigens shared between tumor tissue and skin (103). Moreover, some of the antigen-specific T cells found in blood samples were also present in autoimmune skin lesions and lung tumors of patients who responded to anti–PD-1 therapy. Interestingly, the authors also showed that skin irAEs were more frequent in patients with complete or partial remission as compared to patients with progressive or stable disease. They also demonstrated that, after lung, skin was the second most similar organ to NSCLC histology and had the second highest proportion of autoimmune adverse effects in these patients (103). The same mechanisms of “shared antigens” recognized by common T cell clones between tumor and anatomical site affecting by irAEs have also been demonstrated for cardiac irAEs (104, 105). Nevertheless, it should be noted that deciphering the association between irAEs and clinical response poses challenges for several reasons: i) a potential “immortal-time bias” arises from the fact that shorter survival corresponds to a lower chance of irAE development. By contrary, patients displaying the most favorable responses to immunotherapies often undergo prolonged treatment, increasing their likelihood of developing toxicities; ii) establishing a definitive diagnosis of immune-mediated toxicities is complicated, especially in patients receiving concurrent treatments such as tyrosine kinase inhibitors or chemotherapy (106); iii) the management of irAEs involving ICI discontinuation and resolutive therapy (steroid usage) may impact the patients responses to ICI; iv) chronic or exceptionally delayed side effects can occur, some of which appear even after the completion of the treatment regimen (5, 106–110). These complexities underscore the interest of evaluating the interplay between irAEs and treatment outcomes. It also raises the question of how to disentangle the desirable anti-tumor effects of anti-PD-1 and anti-CTLA-4 therapies from the adverse autoimmune reactions, ultimately striving for a more precise and safer immunotherapeutic approach.

The management of irAEs currently relies primarily on immunomodulators such as glucocorticoids administration associated or not with the temporary or definitive discontinuation of treatment, especially for high severity grade toxicities (grade ≥2) (71, 108, 110) - discontinuation include mostly gastrointestinal, pancreatic, endocrine, hepatic, respiratory, skin, neurological and cardiomuscular disorders. However, steroid treatment approach may be associated with long-term side effects, various harmful effects affecting multiple organs, increased susceptibility to opportunistic infections and emergence of steroid resistance mechanisms. Additionally, some patients exhibit contraindications to steroid use (i.e., diabetes, metabolic syndromes). A significant consideration is also the fact that immunosuppressive glucocorticoids may exert an effect on the TME that contradicts the intended impact of ICI. In murine models, exogenous glucocorticoids injection inhibits the efficacy of immunogenic chemotherapy (111). Another study in lung cancer patients shows that corticosteroid co-administrated with chemotherapy negatively impacts the density of TLS and the formation of germinal centers in the tumor (53). Indeed, multiple parameters regarding steroid administration, including time of exposure, dose, temporal administration during ICI treatment, can differentially impact anti-tumor responses (112).

## Non-steroidal strategies for irAEs management

In addition to other chemical immunosuppressive agents (mycophenolate mofetil, calcineurin inhibitors, cyclophosphamide, methotrexate), various biological drugs have been employed in severe or steroid-refractory case, including cytokines blockers or immune cells depleting mAbs (106, 108, 113). It is important to emphasize that to optimize the management of various toxicities and personalize the selection of immunosuppressive agents, it is essential to thoroughly understand the specific immunological pathways underlying irAEs that can be targeted. As an example, for T-cell-dominated irAEs, anti-IL-6 (tocilizumab), -IL-1 receptor (anakinra or canakinumab), IL-12/23 or IL-17 therapies should be considered. IrAEs associated with neutrophilic and monocytic patterns could be targeted with anti-TNFα (infliximab) strategies. Anti-B-cell strategies [i.e., anti-CD20 mAbs (rituximab, obinutuzumab, ofatumumab), or blockade of B-cell activating factor (belimumab, anti-BAFF mAb)] might be used for irAEs in which B cells and plasma cells are key factors. Nevertheless, caution consideration should be taken regarding the potential impact of this type of depletion on the intricately woven anti-tumoral immune mechanisms. Specifically, the depletion of B lymphocytes can induce the disappearance of TLS formed in autoimmune and infectious diseases (114–116). Moreover, in melanoma cases, B cell depletion has been associated with a decrease in the infiltration and activity of macrophages and cytotoxic T lymphocytes within the TME (117). Consequently, a comprehensive phenotypic analysis of B cell subsets, whether they are predictive markers or effector cells, within immune-related irAEs is imperative.

## Screening of B-cell related early biomarkers of irAEs

In connection with B cells and humoral responses, the presence of preexisting autoantibodies before treatment has been suggested as an early biomarker for the development of ICI-induced autoimmunity, although it remains a topic of controversy. A large number of clinical studies have demonstrated association between irAEs and seropositivity for various autoantibodies, most of them commonly reported in autoimmune diseases (118, 119). A meta-analysis from systemic literature review (515 studies included, excluding studies including patients with pre-existing autoimmune diseases) showed that autoantibody positivity is high in irAEs involving endocrine organs, skin, and muscle but lower in irAEs affecting other organs (119). Autoantibodies are present in about 50% of patients with ICI-associated endocrinopathies, 47% of patients with thyroid diseases are positive for any thyroid autoantibody, and 48% of patients experiencing diabetes mellitus (DM) were positive for any DM-associated autoantibodies (glutamic acid decarboxylase antibodies, anti-IA2, anti-islet, anti-insuline, anti-ZnT8…). Moreover, antibodies directed against BP180 (a glycoprotein found at the dermo-epidermal junction) are found in more than 50% of patients with skin irAES. Finally, autoantibodies were also common in patients with the triad myositis/myasthenia/myocarditis with 49% of patients exhibiting striational antibodies, 40% anti-acetyl choline receptor antibodies, and 27% myositis-associated antibodies (119).

Some studies report a correlation between preexisting autoantibodies and irAEs occurrence in patients (120–123). Utilizing a high-throughput array technology that screens autoantibody reactivity against 162 autoantigens, recent research suggests employing a pre-existing autoreactivity score as a predictive marker for the development of irAEs of grade 2 or higher, irrespective of the type of toxicity or the type of autoantigens recognized (124). However, others failed to demonstrate this association (119, 125). These conflicting results are probably due to a lack of standardization of autoantibodies detection methods, as well as a focus solely on antibodies previously implicated in autoimmune diseases. It is noteworthy that some studies argue that, more than baseline autoreactivity, fold change in autoantibodies concentrations during the treatment is predictive of the occurrence of certain irAEs (125, 126).

## Alternative therapeutic strategies for patients with high risk of irAEs: preserving anti-tumor response and reducing autoimmune side effects

Among the solutions that can be considered to treat patients at high risk of developing irAEs, we can mention the implementation of anti-checkpoint antibodies with reduced toxicity (127). Notably, anti-T cell immunoreceptor with Ig and ITIM domains (TIGIT) antibodies – TIGIT is an inhibitory immune checkpoint notably expressed on T cells, NK cells and Tregs - were found to be generally well tolerated when administered as monotherapy or in combination with PD-1/PD-L1 inhibitors (128). One possible explanation for the milder toxicity of anti-TIGIT antibodies might stem from the fact that this immune checkpoint is not involved in T-cell priming, unlike CTLA-4 molecule. Reactivation of a broader repertoire of T cells by anti-CTLA-4 mAbs is more prone to trigger the activation of self-reactive clones. Moreover, whereas genetic deletion or blockade of inhibitory immune checkpoint LAG-3 (expressed on exhausted T cells infiltrating the tumor and which is also not involved in T cells priming) exacerbates type 1 diabetes in non-obese diabetic mice, LAG-3 deficiency itself does not lead to autoimmunity in non-autoimmune-prone mouse strains (129). A cross-comparison study using survival data and safety profiles from two clinical trials was conducted to assess the impact of LAG-3/PD-1 inhibition (relatlimab–nivolumab therapy) versus CTLA-4/PD-1 inhibition (ipilimumab–nivolumab) in untreated advanced melanoma patients (130). The results showed that, while demonstrating overall similar therapeutic efficacy, relatlimab–nivolumab had fewer grade 3 or 4 irAEs compared to nivolumab–ipilimumab. These encouraging observations pave the way for the utilization of this specific combination of anti-immune checkpoint antibodies, maintaining similar effectiveness but exhibiting a superior safety profile (131).

## Concluding remarks

Immuno-oncology and autoimmunity are often considered as two distinct fields. However, their close association in cancer patients, both during the tumorigenesis or throughout anti-tumor treatment in the era of anti-immune checkpoints inhibitors, highlights the potential to learn mechanistic aspects from each other. Notably, the comparison of self-reactivity patterns in cancer-associated patients with those commonly associated with the corresponding autoimmune pathology might shed new light on self-antigens able to trigger a humoral and/or cellular response in a cancer context. This could open therapeutic perspectives for the development of therapeutic monoclonal antibodies targeting tumor antigens.

Lastly, the bidirectional association between the two pathologies has to be revisited in the context of autoimmune side effects induced by immunotherapies. An urgent need exists to deeply investigate the immunological mechanisms—especially those related to B lymphocytes, which may have been less evaluated—both specific and common across various types of irAEs to better prevent and manage them effectively. This could involve employing more targeted approaches to manage irAEs, thereby reducing dependence on broad immunosuppressive glucocorticoids. Additionally, this exploration could uncover novel combinations of immune checkpoint inhibitors (or alternative immunotherapies) that mitigate adverse events without compromising the effectiveness of immunotherapy.

## Author contributions

SSo: Writing – original draft. GP: Writing – original draft. IC: Writing – review & editing. P-EJ: Writing – review & editing. CS-F: Writing – review & editing. WF: Writing – review & editing. SSi: Writing – original draft.
